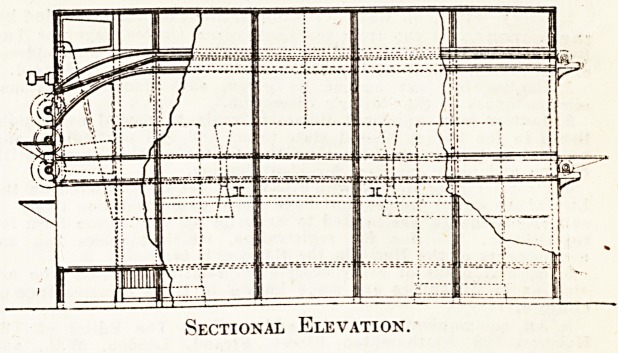# Marr's Patent Drying Machine

**Published:** 1912-11-23

**Authors:** 


### Marr's Patent Drying Machine.

THE illustrations given below show the drying machine in use at the Leeds General Infirmary. This machine consists mainly of two contiguous chambers formed of steel sections bolted together, the larger being the drying chamber and the smaller the heater and fan chamber. The division between the two chambers stops short of the floor and the cover of the machine, forming openings through which the air passes from the fan chamber into the drying

PLAN.

chambers through the top openings, and from the drying chamber back to the fan chamber through the lower openings. The circulation of warm air downwards through the wet clothes and upwards over the air heaters is continuous, and as moisture is evaporated from the wet clothes it is exhausted by a small fan. This arrangement of air circulation makes the machine highly efficient and the steam consumption very small.

In the drying chamber are two pairs of endless chains, one above the other, operated mechanically, to which are bolted at intervals of 4 inches steel bars carrying auto-

END ELEVATION.

matic clips arranged to take small or large articles. The attendants place the wet clothes in the clips at the feed end of the machine, through which they are moved mechanically (the speed of which can be varied to suit the articles being dried) to the opposite end of the machine, where the clips automatically release them dry to drop into suitable receptacles, no attention being necessary at the delivery end except to empty the receptacles of dry goods. The clips and steel bars are galvanised to protect the clothes. Large bed sheets, blankets, and bed covers are folded lengthwise in the centre and the fold placed in the clips, one such article being placed upon each clip bar and held by the eight clips thereon, the drying being done as fast as the attendants can fold and clip the clothes. Smaller articles, such as towels, aprons, nurses' dresses, etc., are placed four on each clip bar, two clips holding each article. The average time the garments are in the drying chamber is forty minutes, during which time they are suspended in open order and subjected to a current of pure warm air, the velocity of which is 300 feet per minute. The illustrations represent a machine 20 feet long in the drying chamber, which contains 132 clip bars in constant use. The capacity of this machine is 20,000 articles per week, and the price of the machine delivered and fixed upon a suitable prepared site is £450 net, exclusive of any driving shafts, pulleys, or belts, steam supply, or drain pipes. The price, however, varies according to the size of the machine, which may be anything from 12 feet to 32 feet long, in multiples of 4 feet, according to the number of articles to be dried per day. The machines

Sectional Elevation.

always work at their maximum capacity, and they can be easily extended at any time to compass more work.

## Figures and Tables

**Figure f1:**
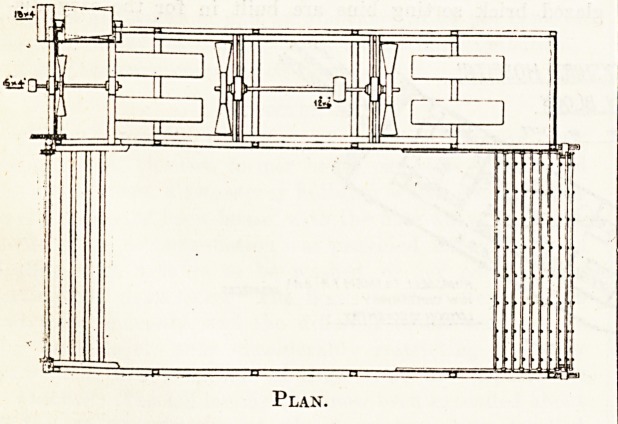


**Figure f2:**
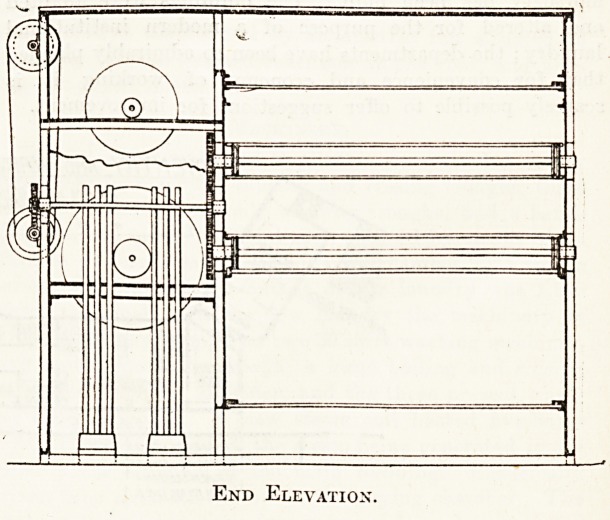


**Figure f3:**